# In silico-designed antimicrobial peptide targeting MRSA and *E. coli* with antibacterial and antibiofilm actions

**DOI:** 10.1038/s41598-024-58039-1

**Published:** 2024-05-27

**Authors:** Hafsa Madni, Hana A. Mohamed, Hana Adel Mohamed Abdelrahman, Carlos André dos Santos-Silva, Ana Maria Benko-Iseppon, Zenaba Khatir, Nahla O. Eltai, Nura A. Mohamed, Sergio Crovella

**Affiliations:** 1 Biological and Environmental Sciences Department, Qatar University, PO Box 2713, Doha, Qatar; 2 Biomedical Research Center, Qatar University, PO Box 2713, Doha, Qatar; 3 Department of Biomedical Sciences, Cesmac University Center, PO Box 57051-160, Maceió, AL Brazil; 4 Department of Biomedical Sciences, University Center Cesamc, PO Box 57051-160, Naceio-AL, Brazil; 5 Environmental Science Center, Qatar University, PO Box 2713, Doha, Qatar; 6 Laboratory Animal Research Center, Qatar University, PO Box 2713, Doha, Qatar

**Keywords:** Biological techniques, Cell biology, Computational biology and bioinformatics, Drug discovery, Microbiology

## Abstract

Antibiotic resistance is a paramount global health issue, with numerous bacterial strains continually fortifying their resistance against diverse antibiotics. This surge in resistance levels primarily stems from the overuse and misuse of antibiotics in human, animal, and environmental contexts. In this study, we advocate for exploring alternative molecules exhibiting antibacterial properties to counteract the escalating antibiotic resistance. We identified a synthetic antimicrobial peptide (AMP) by using computational search in AMP public databases and further engineering through molecular docking and dynamics. Microbiological evaluation, cytotoxicity, genotoycity, and hemolysis experiments were then performed. The designed AMP underwent rigorous testing for antibacterial and antibiofilm activities against Methicillin-Resistant *Staphylococcus aureus* (MRSA) and *Escherichia coli* (*E. coli*), representing gram-positive and gram-negative bacteria, respectively. Subsequently, the safety profile of the AMP was assessed in vitro using human fibroblast cells and a human blood sample. The selected AMP demonstrated robust antibacterial and antibiofilm efficacy against MRSA and *E. coli*, with an added assurance of non-cytotoxicity and non-genotoxicity towards human fibroblasts. Also, the AMP did not demonstrate any hemolytic activity. Our findings emphasize the considerable promise of the AMP as a viable alternative antibacterial agent, showcasing its potential to combat antibiotic resistance effectively.

## Introduction

The imprudent utilization of antibiotics across human healthcare, veterinary practices, agricultural settings, and environmental contexts has significantly contributed to the emergence and escalation of antimicrobial resistance (AMR) against crucial antibiotics^1–4^. Consequently, the imperative for developing innovative drugs impervious to microbial adaptation has become urgent due to the waning efficacy of existing antibiotics. The World Health Organization (WHO) has underscored the alarming ease with which pathogens are developing antibiotic resistance, portraying a striking and persistent trait among bacteria^5^. This underscores the critical need to develop new molecules endowed with antibacterial activity to supplant currently employed antibiotics.

Among the array of suggested molecules, natural antimicrobial peptides (AMPs) are gaining substantial recognition in research. AMPs are diminutive, biologically active proteins present in both prokaryotes and eukaryotes^5^. Typically composed of 12–50 amino acids, AMPs can elicit immune responses against various pathogens, including bacteria, fungi, and viruses^6^. However, these AMPs have drawbacks such as a short half-life, susceptibility to protease degradation, and cytotoxicity to host cells^7^. Leveraging computational methods can augment the selectivity of AMPs for specific targets and optimize their activity. Databases like the Database of Antimicrobial Activity and Structure of Peptides (DBAASP) offer invaluable insights for the in silico design of synthetic AMPs, encompassing peptides evaluated for antimicrobial activity against specific targets^8^.

Plants emerge as exceptional candidates for exploring antimicrobial bioactive molecules. Evolving under challenging conditions and constant threats from pathogens, plants have developed diverse defense mechanisms and structures to counteract infections, including those caused by microorganisms^9^. Plant antimicrobial peptides (PAMPs) arise from these chemical defenses, produced by plants to repel infections and impede their propagation. Typically dispersed throughout the entire plant, from roots to flowers, PAMPs foster plant growth and also provide protection against diseases in both plants and humans^9^. Through the integration of bioinformatics and peptide engineering techniques, PAMPs can be synthesized to maximize their benefits while minimizing drawbacks^10^.

In this study, utilizing advanced in bioinformatics and peptide engineering techniques, we strategically crafted a synthetic peptide with precision and care. The primary objective was to explore and evaluate its antimicrobial activity against specific targets. This involved a comprehensive analysis of the peptide's structure, properties, and potential applications, integrating cutting-edge computational tools to design an effective and targeted solution against microbial threats.

The specific objectives encompassed evaluating the impact of the synthetic AMP sequence on the growth and reproduction of planktonic and biofilm forms of *Escherichia coli* (*E. coli*) and Methicillin-resistant *Staphylococcus aureus* (MRSA). Additionally, the in vitro effects of this synthetic AMP on human cells were investigated in terms of cytotoxicity and genotoxicity. This study marks the inaugural exploration of the antibacterial and antibiofilm effects and in vitro impact of this peptide, providing valuable insights into its potential as a novel therapeutic agent.

## Materials and methods

### AMP synthesis and characterization

We initiated our study by selecting AMP sequences from publicly available repositories such as the Collection of Antimicrobial Peptides (CAMPs), CAMPSign, and ClassAMP^11^. These AMP sequences served as probes for identifying homologous peptides of different eukaryotic species, provided their genomes or transcriptomes were accessible in online repositories^12^. Subsequently, we designed AMP analogs based on the mature AMP sequences obtained in the previous step. Our design criteria included targeting peptides of approximately 10–34 amino acids in size, possessing a cationic surface charge, and showing potential for antimicrobial and therapeutic activity.

#### Molecular modeling

The structural modeling of the AMP was conducted using the ColabFold platform online, leveraging the available version of AlphaFold2 within a Google Colaboratory notebook environment. The AMP sequence of interest was submitted to the tool, which utilizes an artificial intelligence algorithm developed by DeepMind for predicting the three-dimensional structure of proteins. Theoretical model validation was carried out to ensure its reliability and utility for future applications. The quality of the modeled AMP structure was assessed using various metrics, including the per-residue predicted local distance difference test (pLDDT) for confidence score and the global structural similarity score (TM-score)^13^, as detailed in the Supplementary material. Consequently, the model obtained was deemed suitable for further molecular dynamics simulations for more detailed functional and interactional studies.

#### Molecular dynamics simulation

The molecular dynamics simulation of the AMP utilized the GROMACS package, version 2019.4 ^14^. Initially, AMP modeled in previous steps were centered in a cubic box, which was then solvated with the SPC (Simple Point Charge) water model. Following solvation, the box was equilibrated with a NaCl solution at a physiological concentration of 0.15 M, where solute molecules were replaced by Na^+^ and Cl^−^ ions, followed by system energy minimization. To maintain the system temperature at 300 K, the NvT ensemble was used, with solute atoms restrained to their initial positions. The LINCS method was applied to constrain bonds involving hydrogen atoms^15^. Atom movements were integrated using the leapfrog algorithm with an integration step of 2 fs. An initial energy optimization was conducted using 50,000 steps of the steepest descent algorithm. All atomistic simulations were performed over a total period of 100 ns, employing the GROMOS 53A6 force field. The molecular dynamics were carried out without constraints, under constant pressure of 1 atm and temperature, providing a suitable environment for observing the dynamic behavior of the AMP over the simulation time.

Finally, NovoPro Bioscience Inc. (Shanghai, China) commercially synthesized the AMP selected through our bioinformatics pipeline. NovoPro Bioscience conducted assessments of AMP purity using reverse-phase high-performance liquid chromatography (HPLC) and subjected the AMP to mass spectrometry for quality control and molecular weight confirmation. NovoPro Bioscience Inc. (Shanghai, China) was chosen after meticulous screening and validation of AMP quality, selected from a series of biotech companies offering similar services.

The sequence of the designed AMP is AFCGGRCRGFRRRLFCTKAC under QU IP Disclosure D2024-0007 (dated 24/01/2024).

### Growth of MRSA and *E. coli* strains

MRSA and *E. coli* strains were acquired from the microbiology bacterial culture stock at the Biomedical Research Centre (BRC), Qatar University. MRSA (ATCC-33591) and *E. coli* (ATCC-29522) strains were cultivated overnight on nutrient agar (Remel, ThermoFisher Scientific, Lenexa, KS, USA), followed by a 24 h period of incubation at 37 °C and adjustment to 0.5 MacFarland Standard, which was measured using DensiCHEK PLUS (bioMérieux, France)^16^.

### AMP activity

Minimal Inhibitory Concentration (MIC) of AMP MRSA and *E*. *coli* was determined as follows. Six serial dilutions of AMP starting from 2000 μg/ml of 160 μl Mueller Hinton broth (MHB, Lioflchem, Roseto degli Abruzzi, Italy) were transferred to the wells (100 µl each) in a 96-well flat-bottom plate ™ (Microtesttm 96 tissue culture plate, Franklin Lakes, NJ, USA) to get concentrations of 2000, 1000, 500, 250, 125, 62.5, 31.25, 15.6, and 7.8 μg/ml^17^. Then, 10 µl of 0.5 MacFarland for each type (MSRA and/or *E. coli*) of the measured strain was added to each well. To assure sterilization, the 96-well plate contained wells with MHB control, MHB with AMP treatment control, and controls for the bacterial strain mixed with MHB to guarantee bacterial growth. 96-well plates were then sealed tightly with a parafilm and placed in an aerobic shaker (Innova 44 Incubator Shaker) for 24 h at 37 °C at a speed of 100 rpm. After this, the MIC of the treatment that effectively eliminated bacterial growth was determined with the naked eye following previously established procedure in our group^16^ and others^18^.

To determine the Minimum biocidal concentration (MPC) for the AMP, 20 μL of a ten-fold serial dilution of the MIC with AMP was spread onto fresh nutrient agar media and incubated at 37 °C for around 18 h. Bacterial colonies were counted and compared to those grown on control nutrient agar, which had 20 μL of a solution composed only of bacteria and MHB^16^.

### Effect of AMP on MRSA biofilm and *E. coli* biofilm

#### MRSA biofilm and *E. coli* biofilm culture

The first step in creating the biofilm was the selection of borosilicate glass beads 3–4 mm in diameter (ISOLAB Laborgeräte GmbH, Singapore). Then, the selected beads were soaked and cleaned with a soap solution; after that, they were washed with ddH2O, soaked in 80% ethanol for 24 h, and then washed properly using sterile water. Bacteria were then grown on these beads to create the biofilm by taking 20 µL of 0.5 McFarland from the bacterial strains (either MRSA or *E. coli*), adding it to 200 µL of nutrient broth in 96-well flat-bottom plates ™, and placing one bead in each well. 96-well plates were then sealed tightly with a parafilm and placed in an aerobic shaker (Innova 44 Incubator Shaker) for 72 h at 37 °C at a speed of 100 rpm. Every 24 h, 200 µL of fresh media was added to replace the consumed media. The beads were then placed in an Eppendorf tube containing 200 µL of nutrient broth and vortexed for 1 min to loosen any attached bacteria. This step was repeated after 24, 48, and 72 h to observe the density of the biofilm formed on the beads. Then, 100 µL of the solution was serially diluted over ten-fold serial dilutions, and 20 μL of the serial dilution was spread onto fresh nutrient agar media and incubated at 37 °C for 18 h. Biofilm colonies were then counted and reported^16^. Generated biofilms were used to investigate the selected AMP's ability to inhibit the MRSA and *E. coli* biofilms.

#### AMP activity against MRSA and *E. coli* biofilm

The antibiofilm activity of AMP on 72 h biofilm cultures was tested by treating the biofilm beads with 2000, 1000, 500, 250, 125, and 62.5 μg/mL concentrations of the selected AMP in 96-well plates. Plates were then tightly sealed with a parafilm and placed in an aerobic shaker (Innova 44 Incubator Shaker) for 72 h at 37 °C at a speed of 100 rpm. The antibiofilm activity of the AMP was determined after observing the wells that contained a transparent solution with the naked eye^16^. Subsequently, the antibiofilm activity of the selected AMP was determined using SEM, as described below.

### Imaging of MRSA, *E. coli*, MRSA biofilm, and *E. coli* biofilm using scanning electron microscopy (SEM)

Beads containing bacteria and biofilms were freeze-dried^19^, fixed to a stub with sticky carbon tape, and then completely spray-sprayed with a 12-nm layer of gold. SEM (ZEISS 1530 Gemini, Carl Zeiss Microscopy GmbH, Germany) was used to examine and photograph the surface of the beads^16^.

### In vitro cytotoxicity, genotoxicity and hemolysis assessment of the AMP

The cytotoxicity and genotoxicity in vitro effect of the AMP was investigated. Human Fibroblasts (ATCC PCS201010, ThermoFischer Scientific, USA) and cells were seeded at a seeding density of 10,000 Cells/well in 96-well plates using Dulbecco's Modified Eagle Medium (DMEM, Gibco, USA). Media was supplemented with 10% Fetal bovine serum (FBS, Gibco, USA), and penicillin streptomycin (Gibco, USA). Cells were incubated overnight (16–18 h) at humidified conditions (5%CO_2_, 37 °C). Adherent cells were then treated with different concentrations of the AMP 7.8125, 15.625, 62.5, 125, 250, 500, 1000, 2000µg/ml, and cells were then incubated at humidified conditions for 24 h. Then conditioned media was collected at different time points 0, 0.5, 1, 3, 6, 24 h and used for measuring Lactate Dehydrogenase (LDH, Thermofischer, USA) following manufacturers’ instructions. Adherent cells were used to measure cell viability using AlamarBlue® (Invitrogen, USA) following manufacturers’ instructions at the following time points 0, 0.5, 1, 3, 6, and 24 h.

We also investigated the genotoxicity effect of the selected AMP. In this part, human fibroblast cells were cultured in 24-well plates at a seeding density of 100,000 cells/well; cells were then allowed to adhere and proliferate overnight (16–18 h). The following day, cells were treated with AMP (500 and 2000 µg/ml; negative and positive controls were included in this part, and cells were treated with DMEM and H_2_O_2,_ respectively. After which, cells were incubated at humidified conditions for 24 h. Following that, the genotoxicity effect of the AMP was measured using oxiselect™ comet assay (Abcam, UK) and following the manufacturer’s instructions. Images were taken using OLYMPUS BX62, at magnification 40×, attached to an OLYMPUS DP73 digital camera. Image analysis was done using the ImageJ application (OpenComet plugin).

Moreover, we tested the hemolytic activity of the AMP using fresh human blood following starndard protocols^20^. Brefily, fresh Human Blood (500 µl) was collected into 2 mL microcentrifuge tubes containing heparin (30 units) and immediately centrifuged at 3700×*g* rpm for 5 min at room temperature. The pellet containing erythrocytes was then washed 3 × 5 min in 1 × PBS at 3700 rpm, RT. Finally, the pellet containing red blood cells (RBC) was diluted with 1 × PBS to obtain 2% RBC suspension and added to an equal amount (250 µL) of serially-diluted (100 µg/mL, 50 µg/mL, 25 µg/mL, 12.5 µg/mL, 6.25 µg/mL) AMPs to be tested. The microtubes were incubated at 37 °C for 1 h. Samples were then centrifuged at 3700×*g* rpm for 5 min and the supernatant (200 µL) was taken from each microtube and added to a LC6-well microplate for the absorption analysis on microplate reader at 450 nm. The positive control (100% hemolysis) consisted of 0.1% Triton x-100 in 1xPBS (pH 7.4), while 1xPBS served as a negative control (0% hemolysis). Each hemolysis activity assessment test was carried out in two replicates and repeated three times.

### Statistical analysis

Obtained results were analyzed and presented as mean ± S.E.M for n experiments with well-defined figure legends. All statistical tests were performed using GraphPad Prism v5. Statistical analysis for AMP effect was determined using two-way ANOVA or one-way ANOVA followed by Bonferroni Post-tests. Statistical significance was noted at *P < 0.05.

### Ethical approval

No studies on human participants or animals are included in this article.

## Results

### Molecular dynamics simulation

**Figure 1:**
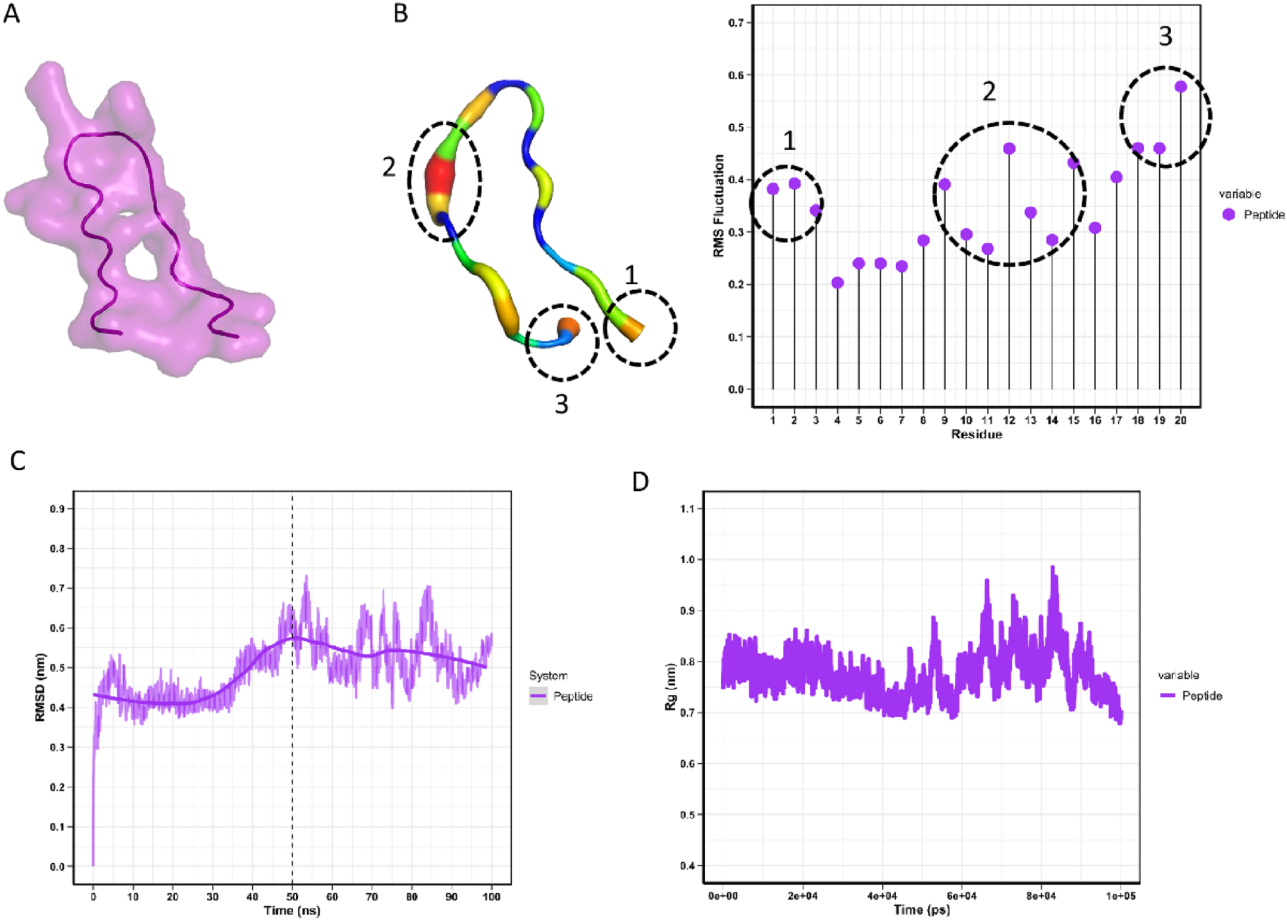
Integrated overview of the AMP molecular dynamics simulation results. (**A**) The three-dimensional conformation modeled by AlphaFold2, illustrating the predicted theoretical structure. (**B**) In Panel (**B**), on the left, the color variation of the model indicates the B-factor of atomic movement, where cooler tones correspond to less mobility and warmer tones to increased atomic mobility; on the right, the RMSF graph highlights residues with greater fluctuations, aligned with areas of intense mobility represented by the B factor. (**C**, **D**) Panels (**C**) and (**D**) display, respectively, the evolution of the RMSD over the simulation time and the behavior of the Rg, both crucial for analyzing the structural stability and conformational dynamics of the AMP during the simulation.

The AMP's Root Mean Square Deviation (RMSD) stabilizes quickly after an initial increase in the first 10 ns, indicating the system's equilibration period. Following this phase, the AMP exhibits minor fluctuations within a range of 0.4–0.7 nm, suggesting that it has reached a state of dynamic equilibrium. The average RMSD after the equilibration period was maintained below 0.7 nm, indicating a stable structure in molecular dynamics simulations. Regarding Root Mean Square Fluctuation (RMSF), it is noted that residues display varying degrees of fluctuation. The residues with higher RMSF values correspond to loops or terminal regions, which generally possess greater mobility (Fig. 1A–D). In terms of the radius of gyration (Rg), at the simulation's outset, the AMP's Rg remains relatively constant, indicating a compact structure. As the simulation progresses, more significant fluctuations are observed, with peaks suggesting the adoption of more expanded conformations. These peaks may be associated with events of partial unfolding or possible loop movements in the structure, increasing the AMP's average. The three-dimensional modeling model of the AMP, homology modelling, sequence coverage and global structural similarity score and per-residue pLDDT have all been assessed (Supplementary Figs. 1–4).

### Antibacterial activity of AMP against MRSA and *E. coli*

**Figure 2:**
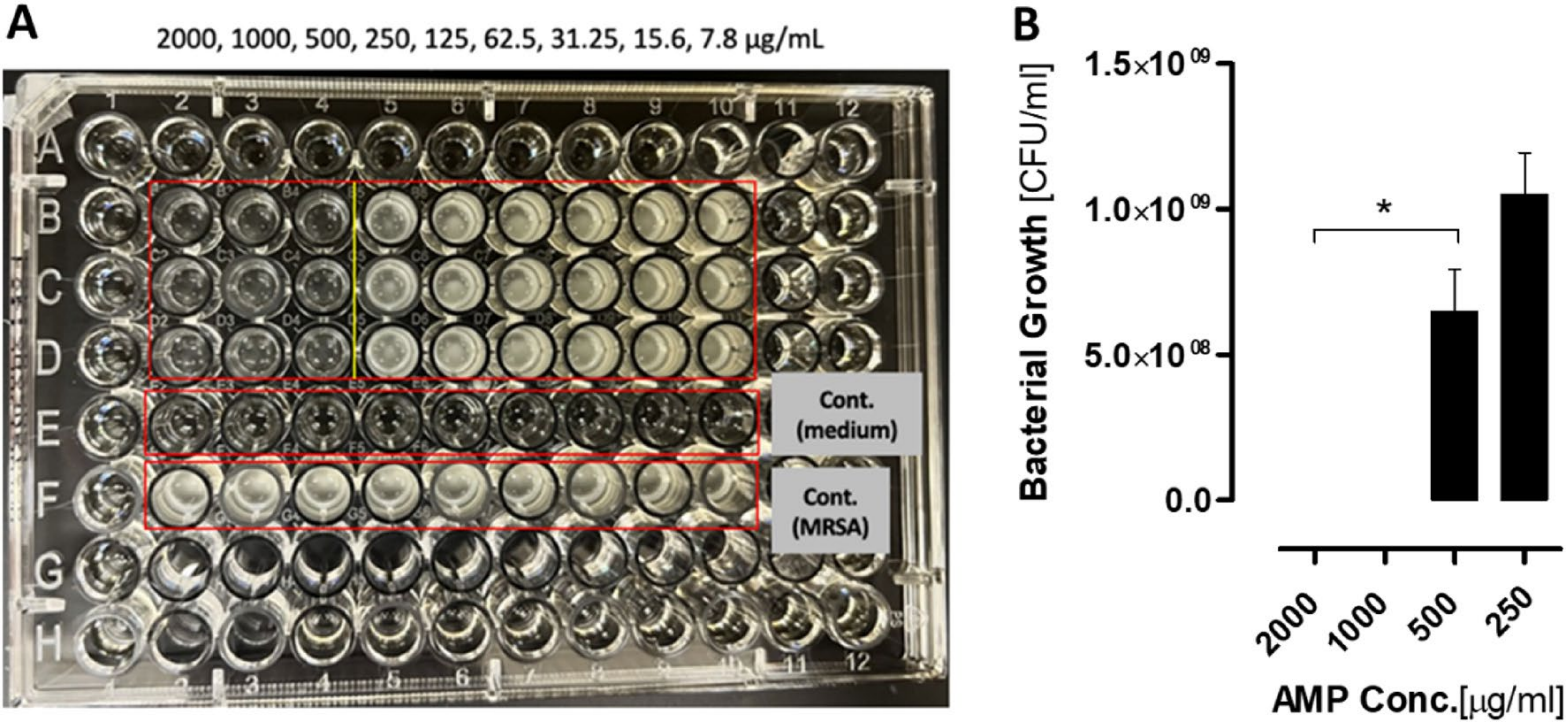
Antibacterial effect of the different concentrations of the AMP on MSRA. (**A**) image of the 96-well plate with the MRSA treated with different AMP concentrations (2000, 1000, 500, 250, 125, 62.5, 31.25, 15.6, 7.8 µg/ml) in wells 2, 3, 4, 5, 6, 7, 8, 9, 10 respectively. Rows B, C and D represent the N = 3 Replicates for each concentration. Turbidity of the treated wells was compared to the MRSA control growth (Row F). (**B**) In this figure MRSA was treated with the following AMP concentrations 2000, 1000, 500, 250 µg/ml, where 500 µg/ml was determined as the MIC, with concentrations lower than 500 µg/ml not affect MRSA growth. Data are mean ± SEM for n = 3. Statistical analysis was performed using one-way ANOVA followed by Bonferroni Post-tests (*P < 0.05).

**Figure 3:**
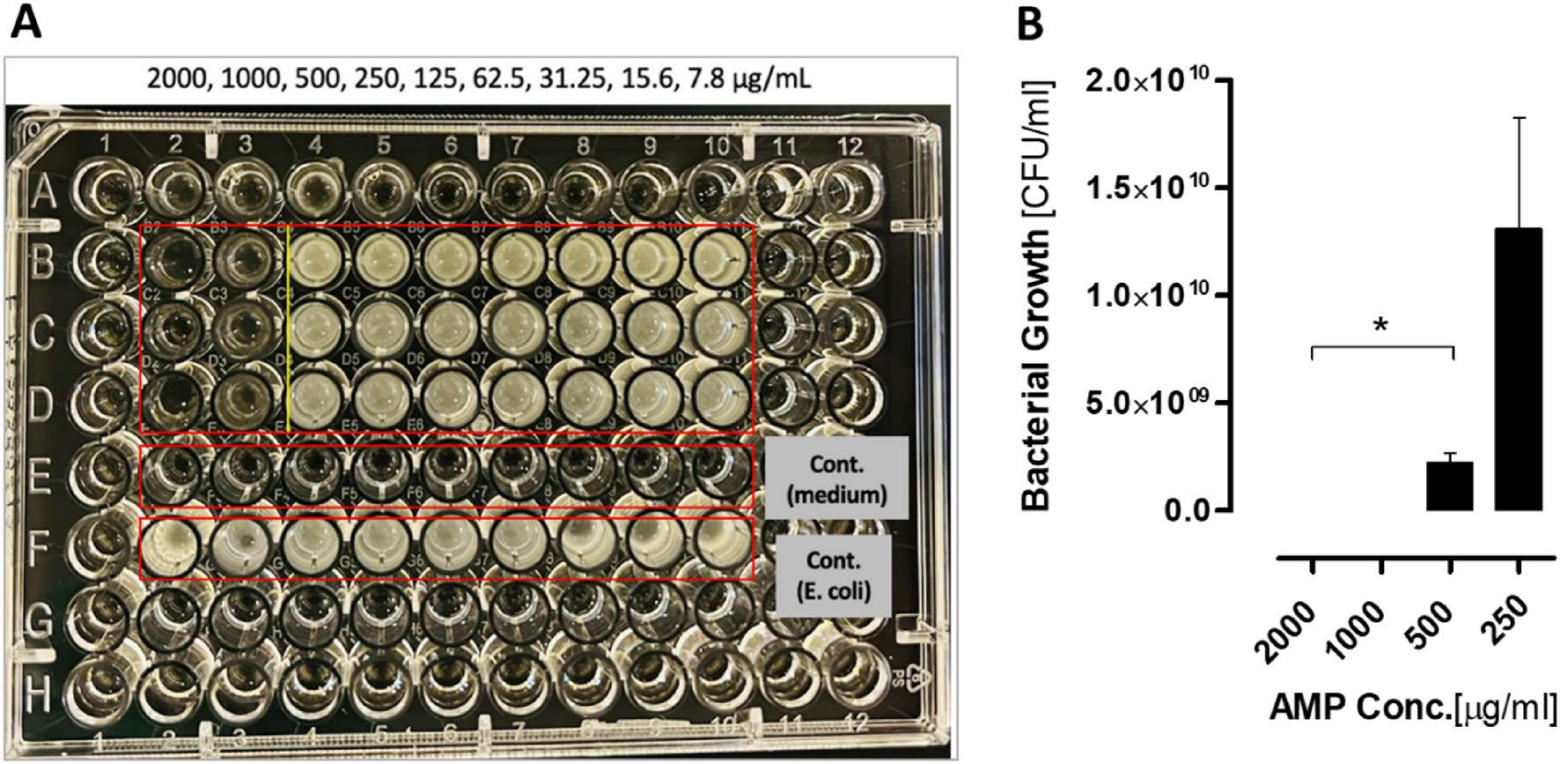
Antibacterial effect of the different concentrations of the AMP on *E. coli*. (**A**) image of the 96-well plate with the *E. coli* treated with different AMP concentrations (2000, 1000, 500, 250, 125, 62.5, 31.25, 15.6, 7.8 µg/ml) in wells 2, 3, 4, 5, 6, 7, 8, 9, 10 respectively. Rows B, C and D represent the N = 3 Replicates for each concentration. Turbidity of the treated wells was compared to the *E. coli* control growth (Row F). (**B**) In this figure *E. coli* was treated with the following AMP concentrations 2000, 1000, 500, 250 µg/ml, where 1000 µg/ml was determined as the MIC, with concentrations lower than 1000 µg/ml not affect *E. coli* growth. Data are mean ± SEM for n = 3. Statistical analysis was performed using one-way ANOVA followed by Bonferroni Post-tests (*P < 0.05).

The antibacterial activity of the selected AMP was evaluated using MRSA and *E. coli* by exposing the bacteria to different AMP concentrations for 24 h. Results showed that the two highest concentrations of the AMP 2000, 1000 μg/ml, demonstrated biocidal effect to the clinical MRSA and *E. coli* strain used in this study, while there was a reduction in bacterial growth at 500 µg/ml AMP concentration with a log reduction of 3 for the MRSA and 2 for the *E. coli* (Figs. 2A, 3A). In turn causing a concentration-dependent change in the color of the solution compared to the other AMP concentrations (250, 125, 62.5, 31.25, 15.6, and 7.8 μg/ml). Moreover, our findings showcased that the minimum inhibitory (MIC) and MBC of the AMP using MRSA and *E. coli* was 500 and 1000 μg/ml, respectively (Figs. 2B, 3B), thus, demonstrating the efficacy of AMP as an antibacterial agent capable of inhibiting both MRSA and *E. coli* strains.

### Bacteria biofilm growth

**Figure 4:**
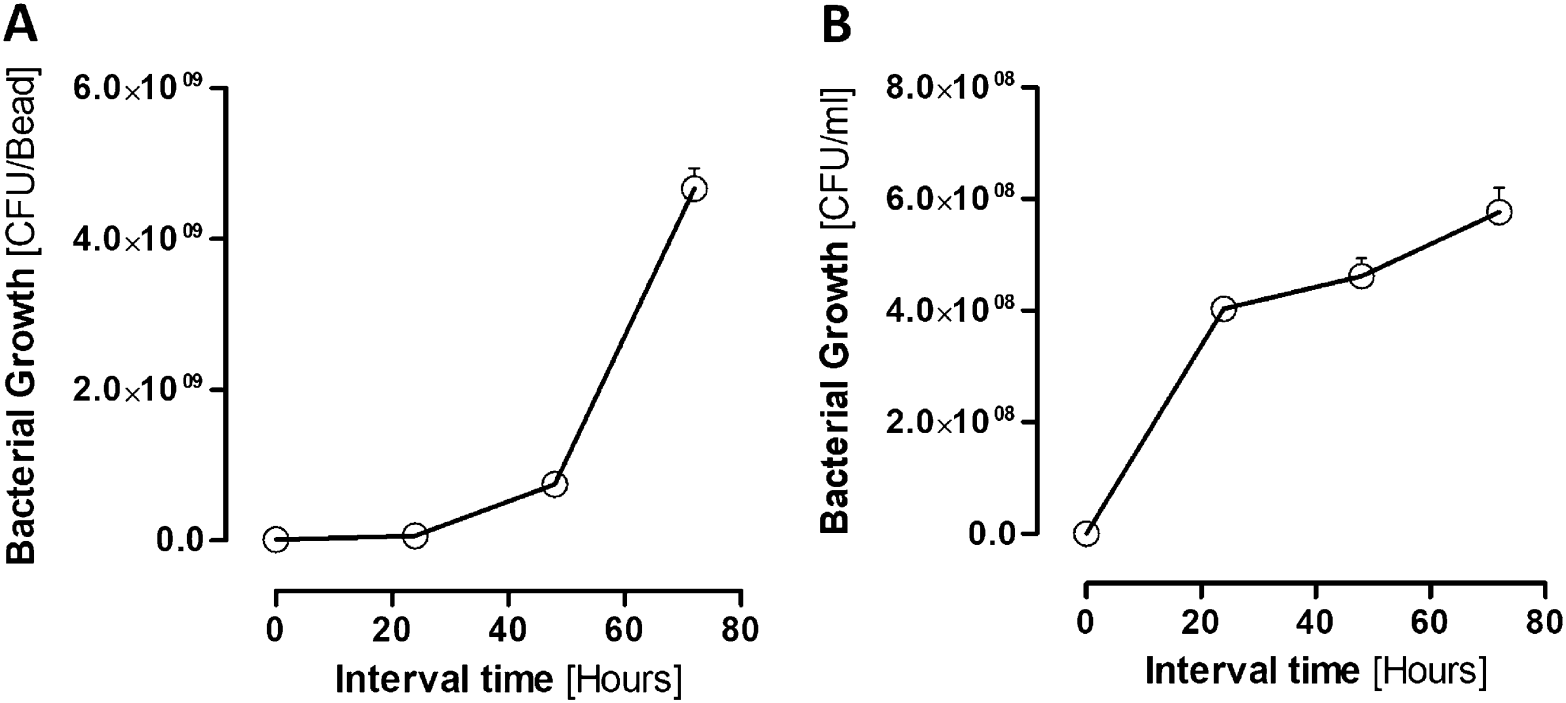
CFU count of (**A**) MRSA and (**B**) *E. coli* Biofilms.

The MRSA and *E. coli* biofilms were developed on the beads’ surface. The biofilm growth was monitored and reported by counting the number of the biofilms formed on the beads at various time intervals, including 0 h, 24 h, 48 h, and 72 h. MRSA biofilm showed exponential growth and continued to increase over time (Fig. 4A), while *E. coli* biofilm was increased up to 24 h, after which it plateaus (Fig. 4B).

### Antibiofilm activity of AMP against MRSA and *E. coli* biofilms

**Figure 5:**
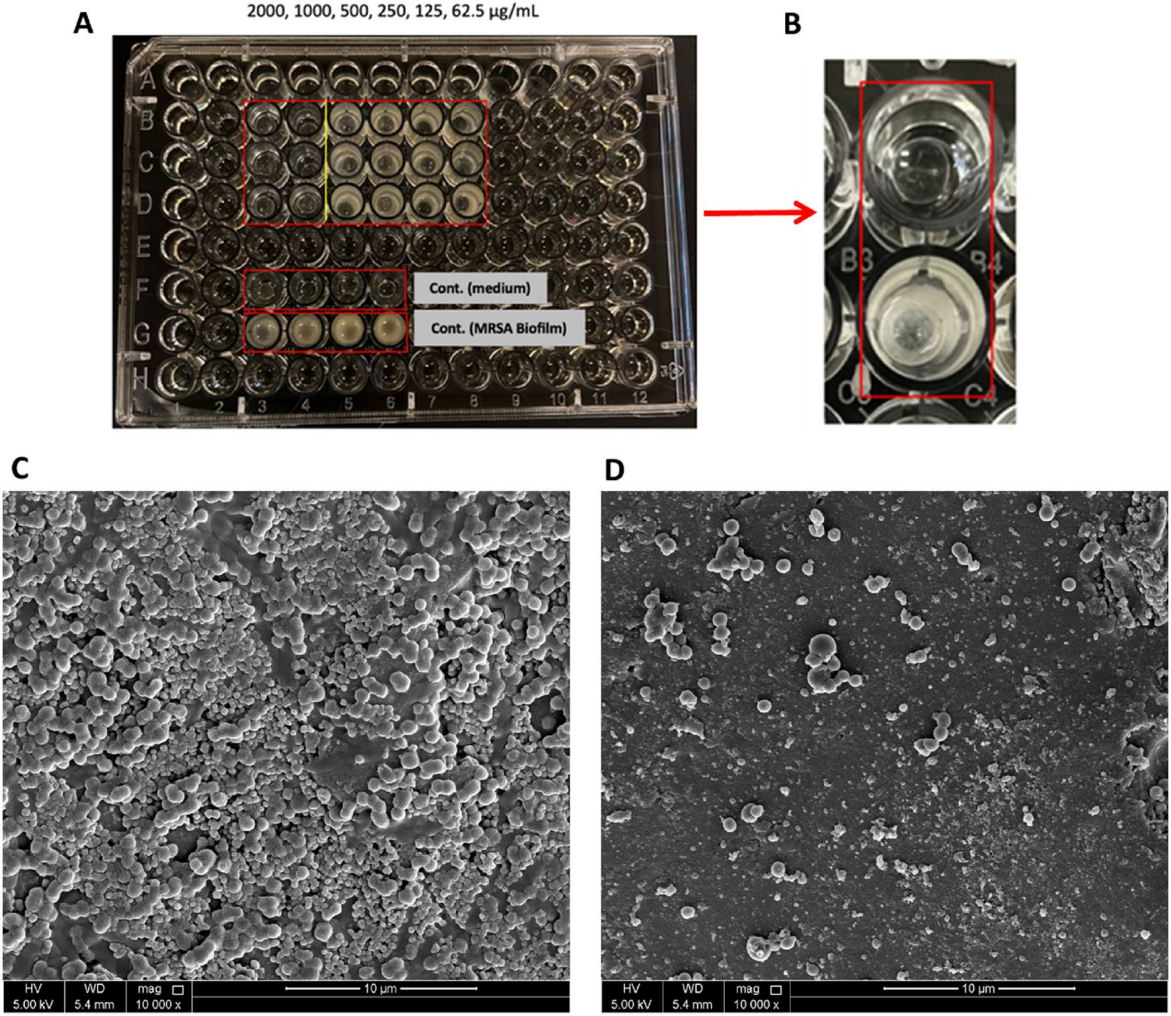
The effect of different concentrations of the synthetic AMP on the MRSA biofilm. (**A**) Image of the 96-well plate with the MRSA biofilms treated with different AMP concentrations. (**B**) Close-up image of a dried bead of the MRSA biofilm treated with 2000 µg/mL treatment and a dried bead of the MRSA biofilm control. (**C**) SEM image of a glass bead surface of the MRSA biofilm control, and (**D**) SEM image of a glass bead surface of the MRSA biofilm treated with 2000 µg/mL for 24h at magnification 10,000×.

**Figure 6:**
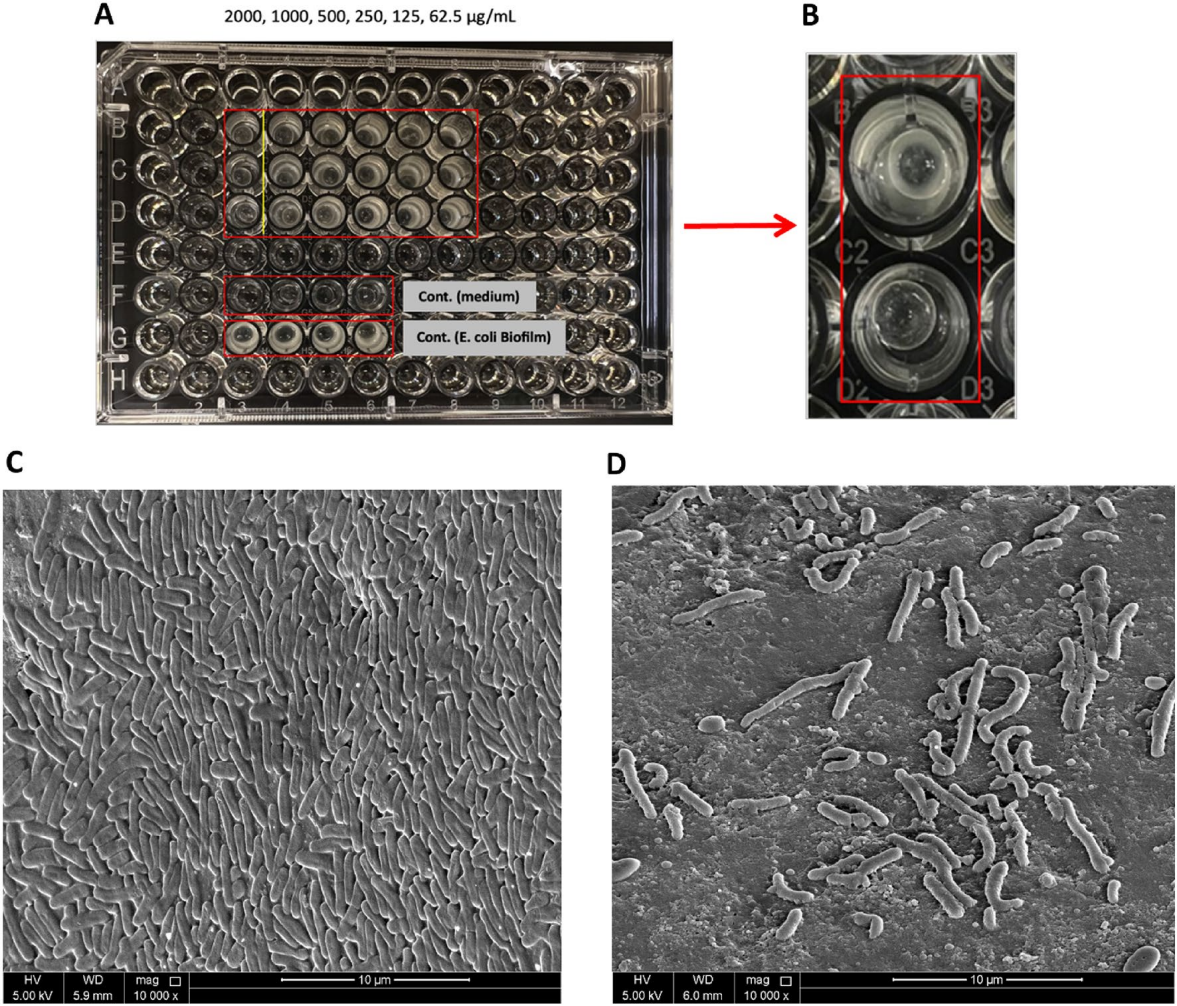
The effect of different concentrations of the synthetic AMP on the *E. coli* biofilm. (**A**) Image of the 96-well plate with the *E. coli* biofilms treated with different AMP concentrations. (**B**) Close-up image of a dried bead of the *E. coli* biofilm treated with 2000 µg/mL treatment and a dried bead of the *E. coli* biofilm control. C) SEM image of a glass bead surface of the *E. coli* biofilm control, and D) SEM image of a glass bead surface of the *E.coli* biofilm treated with 2000 µg/mL for 24h at magnification 10,000X.

The effect of different concentrations of the AMP on the MRSA and *E. coli* biofilms was investigated, with results demonstrating that the first two concentrations of the AMP (2000,1000 μg/ml) were able to eliminate the MRSA biofilm (Fig. 5). On the other hand, only the highest concentration, 2000 µg/ml, could inhibit the *E. coli* biofilm (Fig. 6). Comparably, the change in the media color in the wells containing the beads was observed and paralleled to the remaining concentrations (Figs. 5A, 6A). Furthermore, results showed that the MIC/MBC for the AMP in the MRSA biofilm is 1000 μg/mL (Fig. 5A). With the highest dose of the treatment (2000 μg/ml), totally inhibiting the MRSA biofilm growth (Fig. 5B). On the contrary, the MIC/MBC for *E. coli* biofilm was 2000 μg/mL (Fig. 6A), with the highest dose of the AMP having the efficacy to inhibit the *E. coli* biofilm growth (Fig. 6B).

The SEM images of the surface of glass beads show the difference in the density of the control group of the MRSA biofilm growth compared to the density of the MRSA and *E. coli* biofilms treated with the highest dose of the treatment (2000 µg/ml) (Figs. 5C,D, 6C,D). The findings show the effectiveness of the AMP in inhibiting the growth of the MRSA and *E. coli* biofilm used in our study.

### In vitro assessment of the AMP

**Figure 7:**
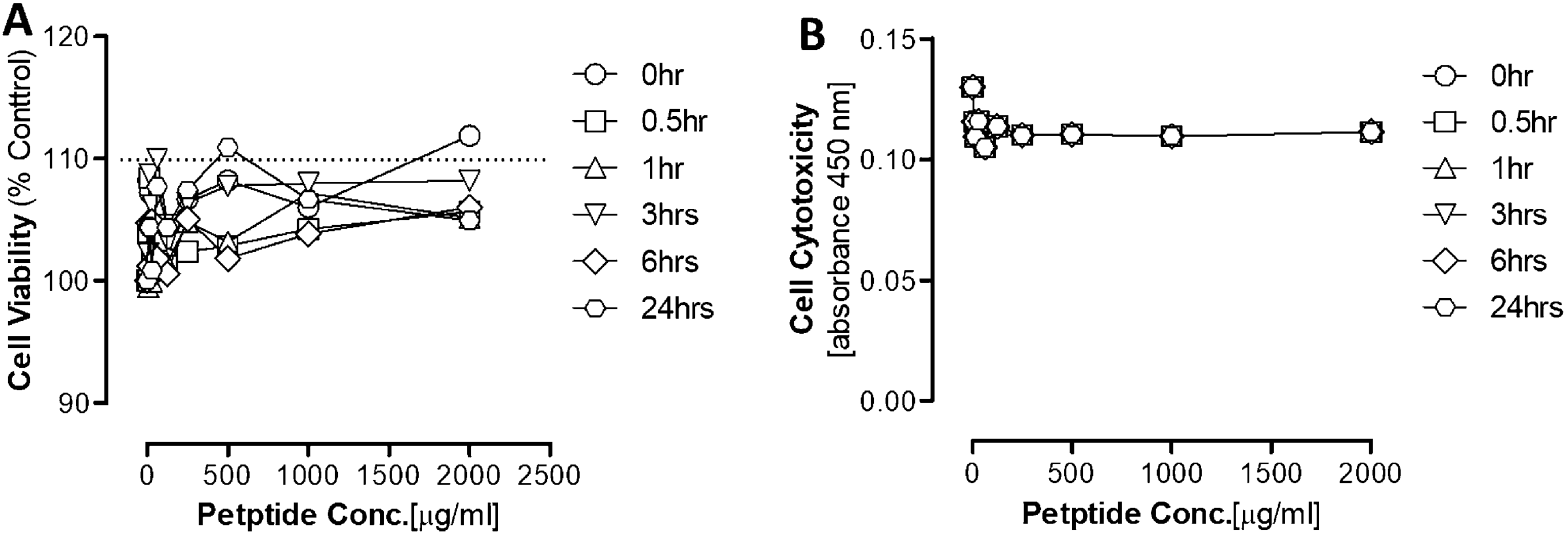
Effect of the AMP on the Fibroblast (**A**) Viability and (**B**) Cytotoxicity. Data are mean ± S.E.M for n = 4: Statistical analysis for effect between different groups was analyzed by two-way ANOVA followed by Bonferroni Posttests. Statistical significance was noted at *P < 0.05.

**Figure 8:**
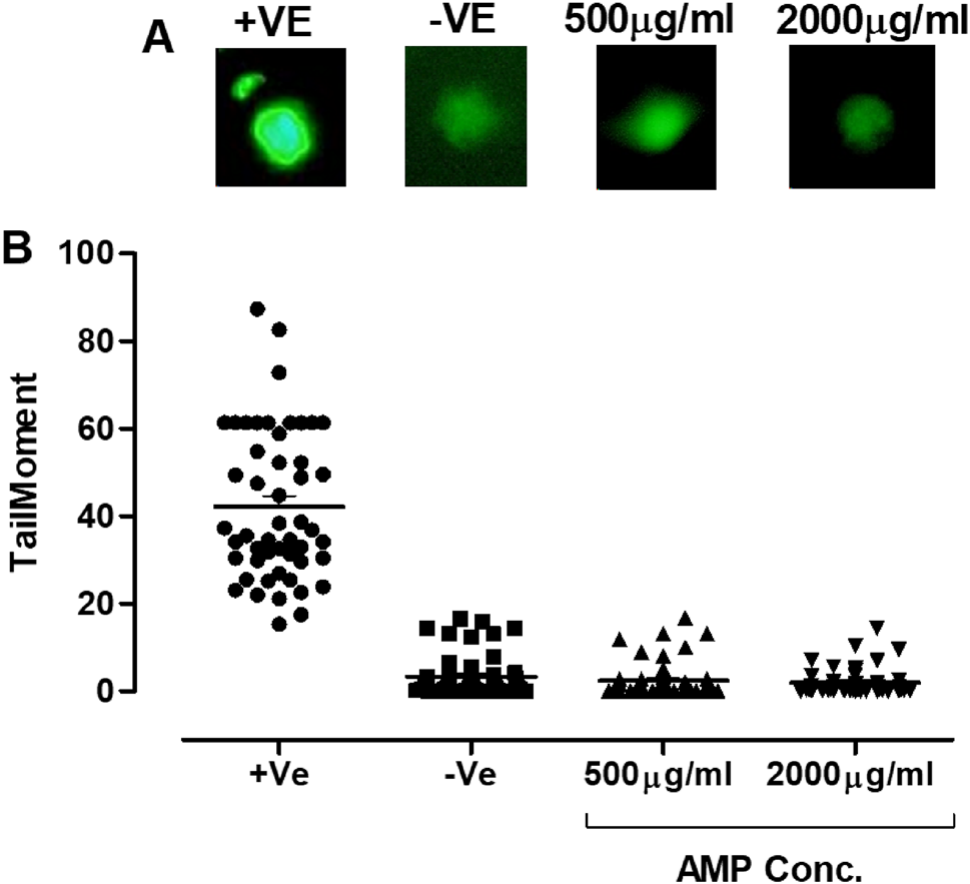
The genotoxicity effect of the AMP on the fibroblast compared to the positive control where cells were treated with H_2_O_2_. (**A**) Representative fluorescent images showing the comet tail of fragmented DNA and extensive DNA migration caused by the +Ve, while treatments with the AMP concentrations 2000, 500 µg/ml did not cause any comet tail and no DNA migration. Images were taken using OLYMPUS BX62, at magnification 40×, attached to an OLYMPUS DP73 digital camera, image analysis was done using ImageJ application (OpenComet plugin). (**B**) Quantification of the extent of DNA migration using tail moments to compare the effect of the different AMP concentration with the +Ve and −Ve.Data are mean ± S.E.M for n = 4. Statistical analysis for effect between different groups was done by one-way ANOVA followed by Bonferroni Posttests. Statistical significance was noted at *P < 0.05.

**Figure 9:**
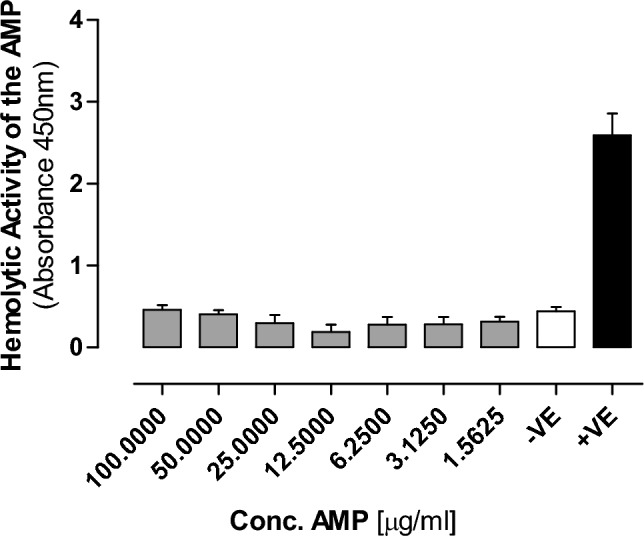
The hemolytic effect of the AMP on the human blood cells (RBCs) compared to the positive control where cells were treated 0.1% Triton x-100 in 1xPBS (pH 7.4). Data are mean ± S.E.M for n = 3. Statistical analysis for effect between different groups was done by one-way ANOVA followed by Bonferroni Posttests. Statistical significance was noted at *P < 0.05.

Results showed that the tested AMP did not affect the cells' viability at all the tested concentrations and time points (Fig. 7A). Moreover, no cellular cytotoxicity was detected using different AMP concentrations (Fig. 7B). Finally, genotoxicity results showed that there was no significant effect caused by the two AMP concentrations showing antimicrobial activity against MRSA and *E. coli* (Fig. 8). Furthermore, data showed that the tested AMP did not have any hemolytic effect on the human RBCs at all the tested concentrations and time points (Fig. 9).

## Discussion

Despite advances in the medical field and efforts to combat bacterial infections with modern techniques, the problem of AMR is increasing^1–4^. According to the World Health Organization (WHO) study, *E. coli* ranks as one of the most dangerous bacteria due to its extremely high resistance to commonly used antibiotics*. S. aureus* bacteria also exhibit significant resistance to multiple antibiotics^21^. Recently, AMPs, which are short sequences of amino acids, have demonstrated their effectiveness against various disease references. Therefore, this study employed bioinformatics and computational techniques to assess the antimicrobial and antibiofilm properties of a bioinspired short antimicrobial peptide on planktonic and biofilm growth of MRSA and E*. coli*. MRSA and *E. coli* are known for their resistance to various antibiotics and treatments, posing significant challenges in the healthcare sector^22,23^.

When considreing the AMP design, the rapid stabilization of the RMSD indicates that the AMP reached an initial conformational equilibrium, suggesting that the adopted structure may be representative of a functional conformation under physiological conditions. The heterogeneous distribution of RMSF along the AMP chain underscores regions of greater flexibility, which may have significant implications for the biological function of the AMP, such as in molecular recognition processes and interaction with other biomolecules. The observed peaks in the Rg fluctuations highlight moments when the AMP may be exploring alternative conformational states. These states could be crucial for biological processes, such as protein folding, protein interactions, and response to environmental stimuli. The dynamic nature of these fluctuations also emphasizes the potential role of structural flexibility in the AMP’s functional adaptation to a complex and variable biological environment.

Our study has uncovered that our synthetic AMP treatment exhibits anti-proliferative properties, effectively curbing MRSA and *E. coli*. Importantly, neither MRSA nor *E. coli* resisted the treatment, indicating its potential to inhibit their growth. However, it is worth noting that the treatment demonstrated greater efficacy in restraining MRSA growth across various concentrations compared to its impact on *E. coli* growth. This variance could be attributed to the gram-negative and positive bacteria structural differences.

For instance, gram-negative bacteria like *E. coli* possess an outer membrane and multiple active efflux pumps, which enhance their resistance to antibiotics compared to gram-positive bacteria^24^. Furthermore, the presence of an electrostatic area in gram-negative bacteria, resulting from the binding of lipopolysaccharides in the outer leaflet with phospholipids in the inner leaflet along with Mg^2+^and Ca^2+^, limits membrane permeability and hinders the entry of many antibiotics, particularly those with a hydrophobic structure, into the bacterial cell^25^. The molecular mechanism underlying the potential action of the AMP in eradicating bacteria is a multifaceted process. Furthermore, due to their cationic nature AMPs exert antibacterial activity by interacting with the negatively charged bacterial cell membranes. Moreover, AMPs possess an amphipathic nature, meaning they have both hydrophobic and hydrophilic regions. When they encounter bacterial membranes, they are believed to initially bind to the surface through electrostatic interactions between their positively charged residues and the negatively charged components of the bacterial membrane, such as lipopolysaccharides in gram-negative bacteria or teichoic acids in gram-positive bacteria. This binding disrupts the membrane's integrity, leading to destabilization and permeabilization. As a result, AMPs can create pores or holes in the bacterial membrane, allowing ions and other essential molecules to leak out, ultimately causing cell death^25^. Additionally, some AMPs may penetrate the bacterial cell interior, interfering with vital intracellular processes such as DNA, RNA, and protein synthesis, further contributing to bacterial elimination^26^. This multifaceted mechanism of action makes AMPs promising candidates for combating bacterial infections and overcoming antibiotic resistance.

Biofilm formation, on the other hand, is a highly organized process executed by microorganisms involving the production of an extracellular polymeric substance that acts as a protective outer layer shielding them from adverse conditions such as temperature and pH fluctuations, dehydration and antimicrobial agents. Among these challenges, bacterial biofilm formation remains a formidable problem in the global healthcare sector. Bacterial biofilms exhibit remarkable drug resistance, rendering them 100–1000 times more resilient than individual bacterial cells. Moreover, these biofilms effectively evade the host's immune responses. Consequently, discovering an effective treatment to eradicate biofilms poses a substantial challenge. Interestingly, our study revealed that the synthetic AMP successfully impeded the growth of MRSA and *E. coli* biofilms without any observed development of resistance. This underscores the treatment's capability to infiltrate and eliminate these biofilms effectively. The mechanisms behind the AMP's biofilm-eliminating action may include penetration and disruption of the biofilm structure or interference with the bacterial cell's biological signals, preventing them from countering external threats^27^. Finally, their biological activity should be assessed for these synthetic AMP to be used in medicine. In this study, we ensured the safety of the selected AMP as it showed no cytotoxicity or genotoxicity at the two AMP concentrations that were shown to be effective against both planktonic and biofilm formation of *E. coli and* MRSA. Furthermore, there was no hemolytic activity detected at all the tested concentrations.

Such results are promising and need to be further assessed using other human cell lines.

## Conclusion

This investigation underscores the pivotal role of a synthetic AMP as a promising agent against bacterial infections and biofilm formation. Our findings unequivocally demonstrate that the employed AMP effectively curtailed planktonic and biofilm growth in MRSA and *E. coli*. Notably, the highest administered dose emerged as a potent strategy for eradicating biofilms formed by both bacterial strains. The comprehensive outcomes of our study substantiate the considerable potential of the synthetic AMP as a therapeutic agent. This potential stems from its unique amphipathic cationic structure, facilitating proficient penetration of negatively charged bacterial cell membranes. This mechanism induces cell disruption, culminating in the demise of bacterial cells. What sets the synthetic AMP in our study apart from others, whether synthetic or natural, is its robust antibacterial and antibiofilm properties and its remarkable non-toxicity to normal human cells. This underscores the imperative need for a meticulous and discerning approach in selecting AMPs based on stringent antibacterial and antibiofilm criteria before advancing them into manufacturing processes. Consequently, these findings underscore the potential of our synthetic AMP as a safe and effective therapeutic intervention against bacterial infections, aligning with the growing demand for innovative solutions in combating antibiotic resistance and biofilm-related challenges in medical settings.

### Supplementary Information

Supplementary Figures.

## Data Availability

The datasets generated and/or analyzed during the current study will be provided by the corresponding authors upon reasonable request.
