# Supplementary material for: In silico-designed antimicrobial peptide targeting MRSA and E. coli with antibacterial and antibiofilm actions
Source: Sci Rep. 2024 May 27;14:12127. doi: 10.1038/s41598-024-58039-1 (PMC11130184; doi:10.1038/s41598-024-58039-1)
Supplement: Supplementary file 1 — Supplementary Figures. [file 41598_2024_58039_MOESM1_ESM.docx]

**In Silico-designed Antimicrobial Peptide Targeting MRSA and *E. coli* with Antibacterial and Antibiofilm Actions**

Hafsa Madni^1^, Hana A. Mohamed^2^, Hana Adel Mohamed Abdelrahman^2^, Carlos André dos Santos-Silva^3^, Ana Maria Benko-Iseppon^4^, Zenaba Khatir^5^, Nahla O. Eltai^2^, Nura A. Mohamed^2*^, Sergio Crovella^6*^.

1. Biological and Environmental Sciences Department, Qatar University, PO Box 2713, Doha, Qatar.
2. Biomedical Research Center, Qatar University, PO Box 2713, Doha, Qatar.
3. Department of Biomedical Sciences, Cesmac University Center, PO Box 57051-160, Maceió-AL, Brazil.
4. Department of Genetics, Federal University of Pernambuco, PO Box 50670-901, Recife, Brazil.
5. Environmental Science Center, Qatar University, PO Box 2713, Doha, Qatar.
6. Laboratory Animal Research Center, Qatar University, PO Box 2713, Doha, Qatar.

**Supplementary Material**

**
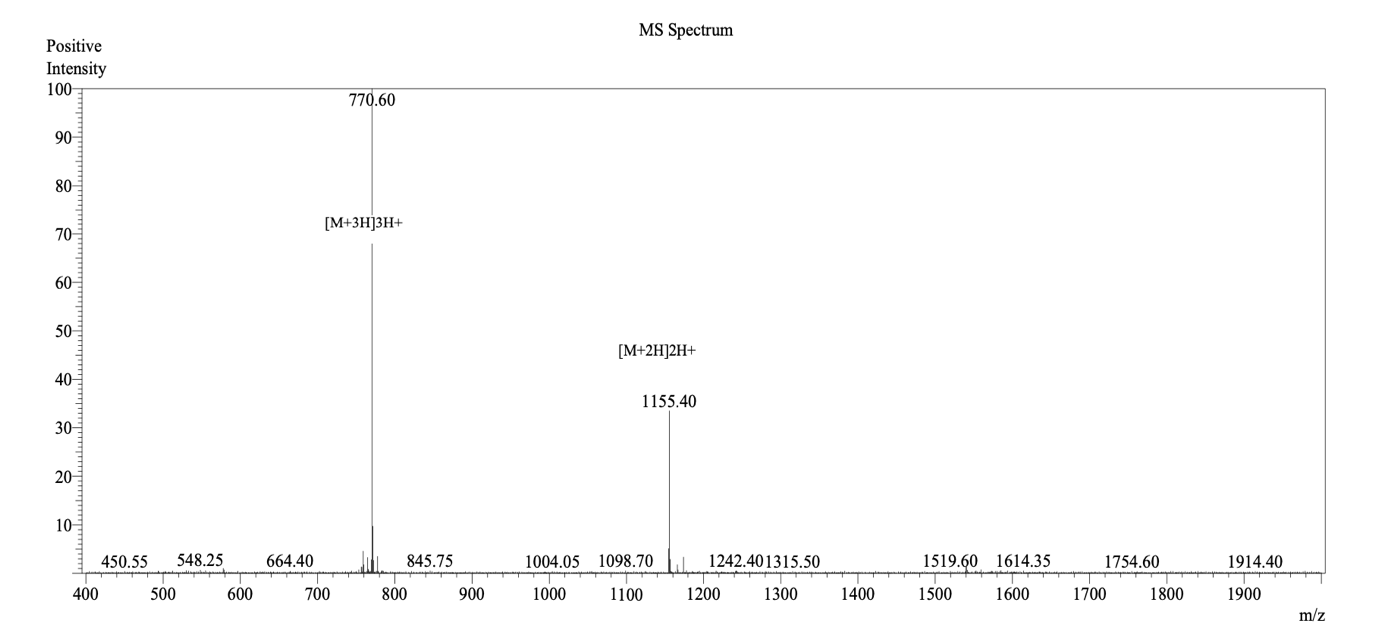
**

**Suplemetary Figure 1:** Mass spectrometry spectrum results for the AMP.


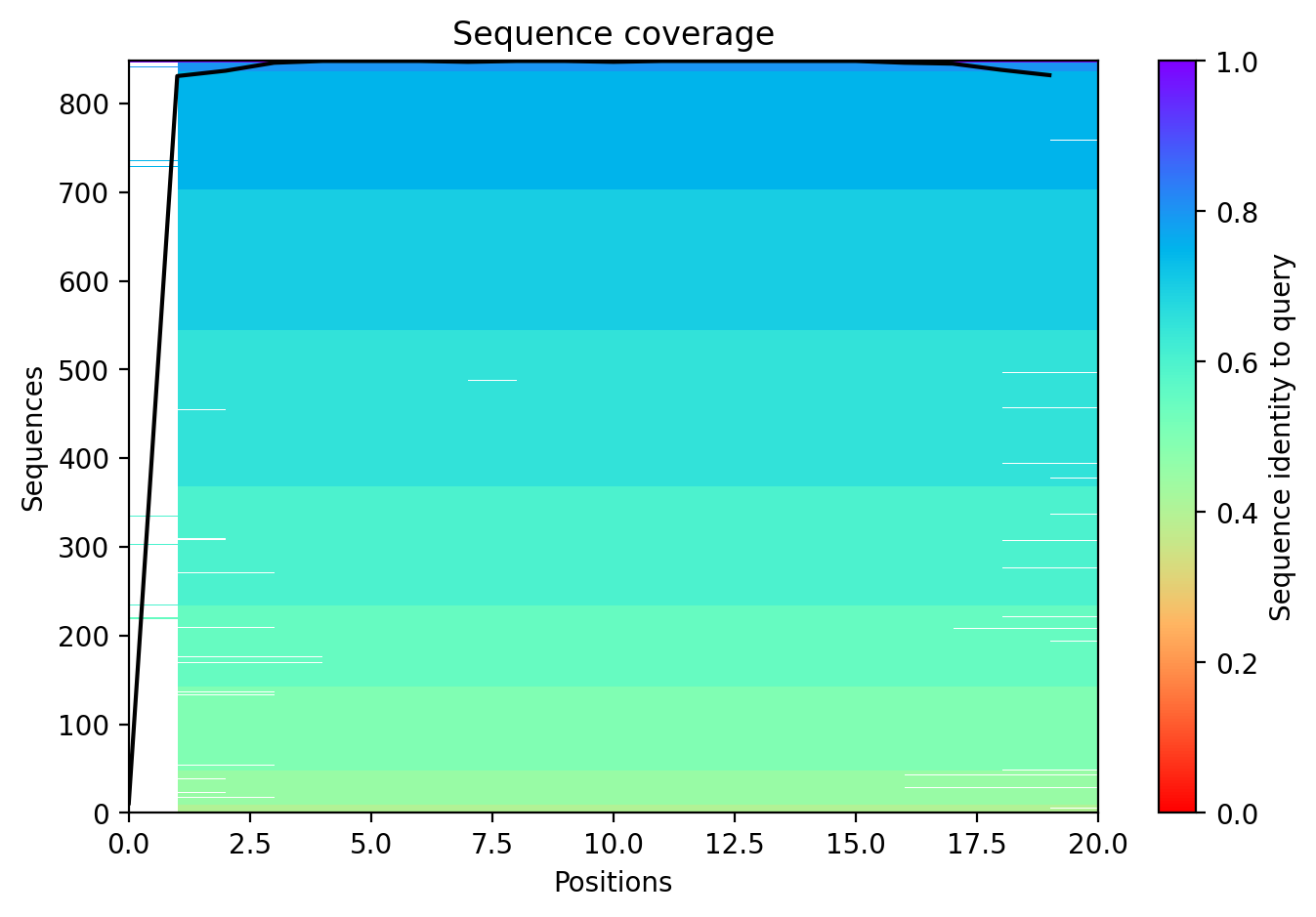


**Suplemetary Figure 2:** The three-dimensional modeling model of the AMP is based on homologous structures and depicts the extent of sequence coverage achieved in the modeling process.

**
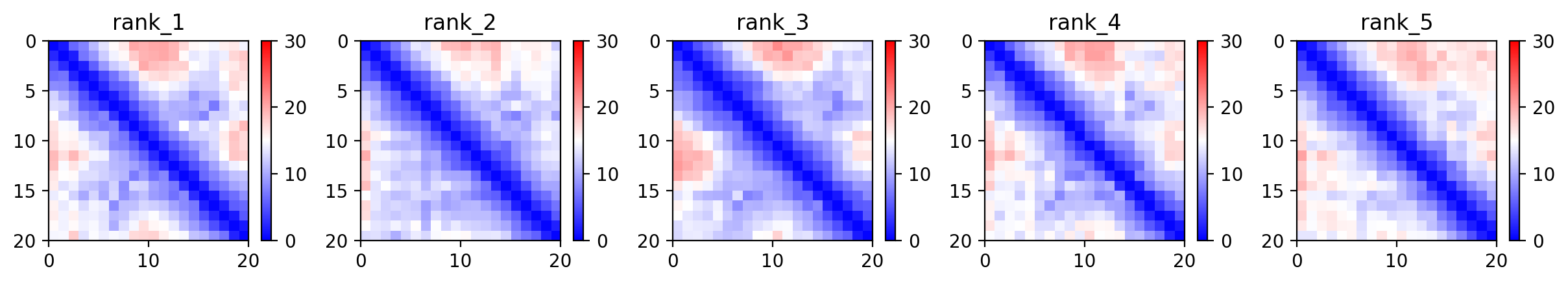
Suplemetary Figure 3:** The global structural similarity score (TM-score) assesses the topological similarity of AMP structures.


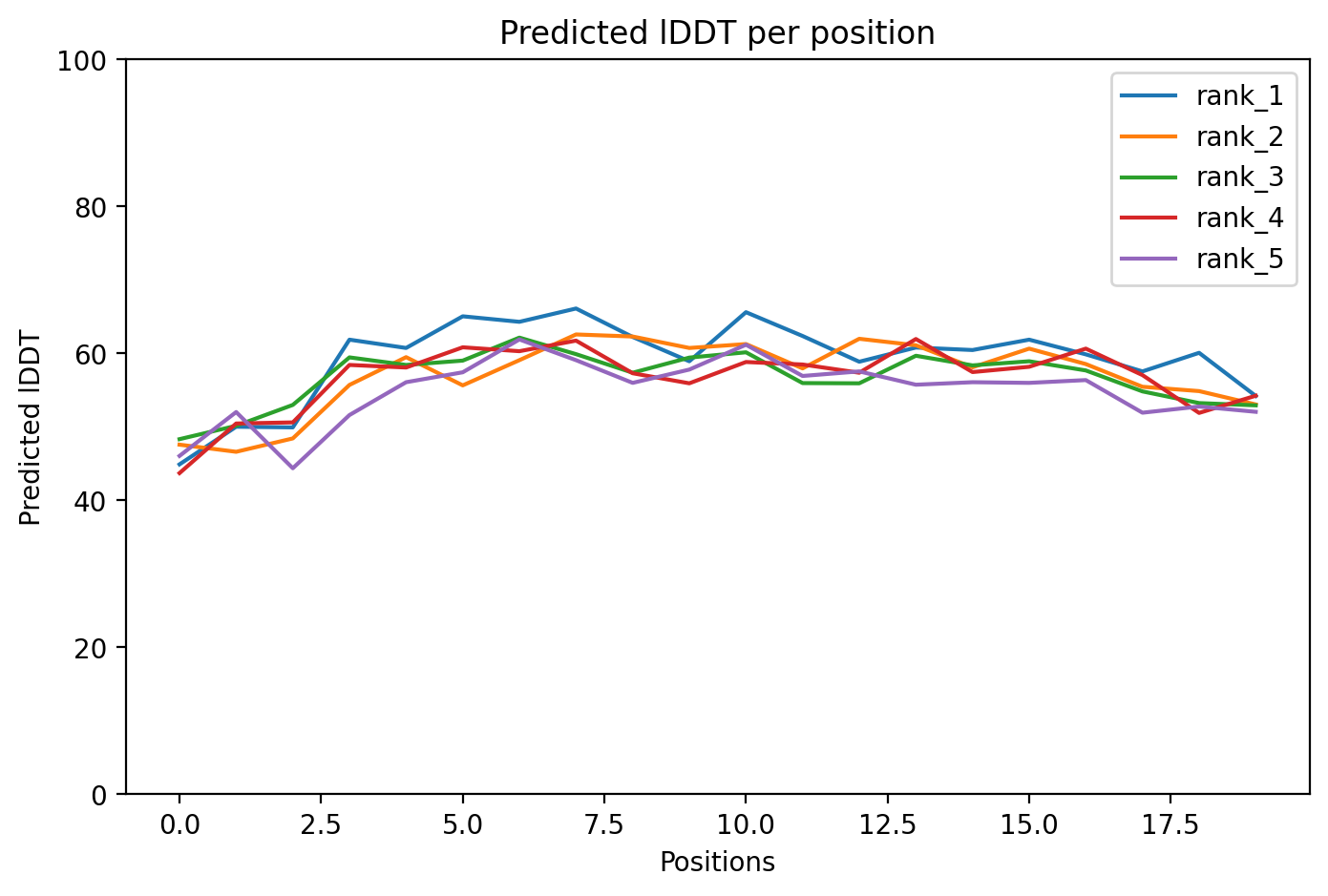


**Suplemetary Figure 4:** Per-residue predicted local distance difference test (pLDDT) scores for each residue of the final model are provided by AlphaFold2. Colors indicate the ranks of predictions.
